# Cytokine systems approach demonstrates differences in innate and pro-inflammatory host responses between genetically distinct MERS-CoV isolates

**DOI:** 10.1186/1471-2164-15-1161

**Published:** 2014-12-22

**Authors:** Christian Selinger, Jennifer Tisoncik-Go, Vineet D Menachery, Sudhakar Agnihothram, G Lynn Law, Jean Chang, Sara M Kelly, Pavel Sova, Ralph S Baric, Michael G Katze

**Affiliations:** Department of Microbiology, School of Medicine, University of Washington, Seattle, Washington USA; Department of Epidemiology, University of North Carolina-Chapel Hill, Chapel Hill, North Carolina USA

**Keywords:** MERS-CoV, Coronavirus, Transcriptomics, Cytokine simulation, Computational topology

## Abstract

**Background:**

The recent emergence of a novel coronavirus in the Middle East (designated MERS-CoV) is a reminder of the zoonotic and pathogenic potential of emerging coronaviruses in humans. Clinical features of Middle East respiratory syndrome (MERS) include atypical pneumonia and progressive respiratory failure that is highly reminiscent of severe acute respiratory syndrome (SARS) caused by SARS-CoV. The host response is a key component of highly pathogenic respiratory virus infection. Here, we computationally analyzed gene expression changes in a human airway epithelial cell line infected with two genetically distinct MERS-CoV strains obtained from human patients, MERS-CoV SA 1 and MERS-CoV Eng 1.

**Results:**

Using topological techniques, including persistence homology and filtered clustering, we performed a comparative transcriptional analysis of human Calu-3 cell host responses to the different MERS-CoV strains, with MERS-CoV Eng 1 inducing early kinetic changes, between 3 and 12 hours post infection, compared to MERS-CoV SA 1. Robust transcriptional changes distinguished the two MERS-CoV strains predominantly at the late time points. Combining statistical analysis of infection and cytokine-stimulated Calu-3 transcriptomics, we identified differential innate responses, including up-regulation of extracellular remodeling genes following MERS-CoV Eng 1 infection and differential pro-inflammatory responses.

**Conclusions:**

Through our genomics-based approach, we found topological differences in the kinetics and magnitude of the host response to MERS-CoV SA 1 and MERS-CoV Eng 1, with differential expression of innate immune and pro-inflammatory responsive genes as a result of IFN, TNF and IL-1α signaling. Predicted activation for STAT3 mediating gene expression relevant for epithelial cell-to-cell adherens and junction signaling in MERS-CoV Eng 1 infection suggest that these transcriptional differences may be the result of amino acid differences in viral proteins known to modulate innate immunity during MERS-CoV infection.

**Electronic supplementary material:**

The online version of this article (doi:10.1186/1471-2164-15-1161) contains supplementary material, which is available to authorized users.

## Background

Middle East respiratory syndrome coronavirus (MERS-CoV) is the etiologic agent of an ongoing respiratory disease outbreak that emerged in Saudi Arabia in 2012. MERS-CoV is most closely related to *Tylonycteris* bat coronavirus HKU4 and *Pipistrellus* bat coronavirus HKU5 [1], highlighting the ever present threat of zoonotic transmission of novel pathogenic coronaviruses. Middle East respiratory syndrome (MERS) resembles acute respiratory disease syndrome (ARDS) caused by severe acute respiratory disease syndrome coronavirus (SARS-CoV) in 2002 and 2003, with some MERS patients exhibiting progressive respiratory distress and renal failure [1, 2]. Despite similarities in overt clinical disease, MERS-CoV is distinct from SARS-CoV in that the virus utilizes a different cellular receptor, dipeptidyl peptidase-4 (DPP4) [3], and exhibits an expanded host cell tropism, readily replicating in a variety of human lung cell types including fibroblasts, microvascular endothelial cells, and type II pneumocytes [4].

Innate immune and pro-inflammatory responses to MERS-CoV remains poorly understood. Human cell culture models of MERS infection have shown a deficiency in interferon (IFN) induction and innate immune responses, which may in part result from multiple mechanisms of MERS-CoV regulation of host antiviral responses. In addition to accessory protein 4a (p4a), the MERS-CoV viral papain-like protease (PLpro) can also block IFN-β induction, as well as downregulate expression of *CCL5* and *CXCL10* pro-inflammatory cytokine genes [5, 6]. Siu and colleagues showed that the block in IFN production is in part the result of MERS-CoV p4a interaction with cellular dsRNA-binding protein PACT that interferes with the activation of RIG-I-like receptors RIG-I and MDA5 [7]. In A549 lung epithelial cells and human bronchus and lung tissue *ex vivo* cultures, MERS-CoV SA 1 failed to induce significant expression differences of *IFNB1* and *TNF* genes relative to mock throughout the 72 hour infection course [8]. The delay of IFN-β expression in response to MERS-CoV was also observed in Calu-3 airway cells [9]. In a separate study, the expression of a panel of interferon-responsive genes, including *DDX58* (encoding RIG-I), *IL1B*, and *CXCL10*, was undetectable in human airway epithelial (HAE) cultures infected with MERS-CoV SA 1, despite efficient viral replication [10]. However, pre-treatment of HAE cells with recombinant IFN-α or IFN-λ suppressed MERS-CoV SA 1 replication, indicating viral sensitivity to innate immune responses [10].

A functional genomics approach revealed MERS-CoV and infectious clone SARS-CoV (icSARS-CoV) activated expression of pathogen recognition receptor genes and pro-inflammatory cytokine genes related to interleukin 17 (IL-17) signaling by IL-17A and IL-17 F cytokines, while differentially regulating antigen presentation pathway gene expression [11]. The human immune response to MERS-CoV appears to be distinct across patients, with increased secretion of IL-17A and IL-23 in bronchoalveolar lavage (BAL) supernatants and increased CXCL10 in serum of patients infected with MERS-CoV [12]. The patient with the poor outcome showed decreased expression of innate immune genes, such as *DDX58*, *IFIH1* (encoding MDA5), *IRF3*, *IRF7*, *IFNA*, and *IFNB1*, which may be the result of host and virus-specific genetic differences. Recent phylogenetic analyses of the complete viral genome from 21 different MERS cases demonstrate the extent of adaptive changes in MERS-CoV since the initial outbreak [13].

To further investigate MERS-CoV regulation of innate immune and pro-inflammatory responses, we utilized an established human airway culture system to examine cellular responses against two genetically distinct MERS-CoV strains, a primary isolate obtained from a Qatari patient treated at a London hospital in September 2012 who later died after prolonged illness in June 2013 (herein referred to as MERS-CoV Eng 1) [2] and MERS-CoV SA 1 (herein referred to as MERS-CoV SA 1), the first virus identified from a fatal case in Saudi Arabia in June 2012 [1]. There are a total of 29 amino acid differences between these two viruses spanning the length of the viral genome, as well as deletions of two amino acids in the nucleocapsid protein of MERS-CoV Eng 1 compared to MERS-CoV SA 1 [14].

## Results and discussion

Using a human airway cell culture model, we sought to understand which specific signaling events would be determinant components of the host response to MERS-CoV infection. We took a genomics-based approach and assessed the whole transcriptome by microarray analysis to 1) topologically characterize the kinetic and magnitudinal changes in the host response elicited by MERS-CoV Eng 1 and MERS-CoV SA 1 and 2) identify contrasting genes between the two viruses related to innate and pro-inflammatory signal stimulation. Utilizing cytokine treatment transcriptomic data sets derived from the same model system, we pursued cytokine signaling events in MERS-CoV-infected Calu-3 cells driving statistically significant contrasting gene expression observed between MERS-CoV Eng 1 and MERS-CoV SA 1. On the basis of the virus-contrasting genes, we predicted STAT3 as a regulator of MERS-CoV-induced host responses, with strain-specific differences in STAT3-mediated gene expression.

### Topological characterization of the host response shows spatio-temporal transcriptomic differences between MERS-CoV strains

Human Calu-3 2B4 cells were infected with one of two different MERS-CoV strains, MERS-CoV SA 1 or MERS-CoV Eng 1, or with icSARS-CoV, and cell lysates were harvested throughout the infection course for microarray analysis. We first characterized topological differences of the whole transcriptome on Euclidean metric space for the collection of 93 samples (mock, MERS-CoV SA 1, MERS-CoV Eng 1 and icSARS-CoV) using recent methods from computational topology, including persistence homology [15, 16]. Many data reduction methods rely on embedding high-dimensional data into two dimensions. Multi-dimensional scaling (MDS), for example, calculates coordinates for a 2-dimensional embedding of the experimental data by minimizing the deviation between embedded and original data (Kruskal stress). Persistence homology, on the other hand, performs computation of topological invariants in the dimension of the original dataset and is, unlike MDS, more stable under perturbation. To assess global kinetic dissimilarities across different CoV infections, we calculated topological differences between MERS-CoV SA 1, MERS-CoV Eng 1 and icSARS-CoV, each grouped throughout all time points and with data set-matched mock samples. The kinetics of the host response refers to the spread of infected samples relative to mock samples over time. There were large topological differences between MERS-CoV Eng 1 and icSARS-CoV (score: 2.42), and between MERS-CoV SA 1 and icSARS-CoV (score: 2.18), and moderate differences between MERS-CoV Eng 1 and MERS-CoV SA 1 (score: 0.29) (Methods and Table 1).

**Table 1:** **Topological assessment of normalized log2 expression for the whole transcriptome and genes restricted to either IFN or pro**-**inflammatory cytokine treatment for MERS**-**CoV Eng 1**, **MERS**-**CoV SA 1 and icSARS**-**CoV conditions**

Topological difference of normalized log2 expression	MERS - CoV SA 1 to Eng 1	MERS - CoV SA 1 to icSARS - CoV	MERS - CoV Eng 1 to icSARS - CoV
**Whole transcriptome**	0.29	2.19	2.43
**Restricted to IFN**-**α and IFN**-**γ stimulated genes**	0.74		
**Restricted to TNF and IL**-**1α stimulated genes**	0.35		

For comparison of topological differences in the host response, we calculated persistence homology bar codes for data set-matched mock and infected samples. The differences between bar codes were calculated using a maximal bipartite graph matching algorithm. For icSARS-CoV we also included 30 and 36 hpi samples.

**Figure 1:**
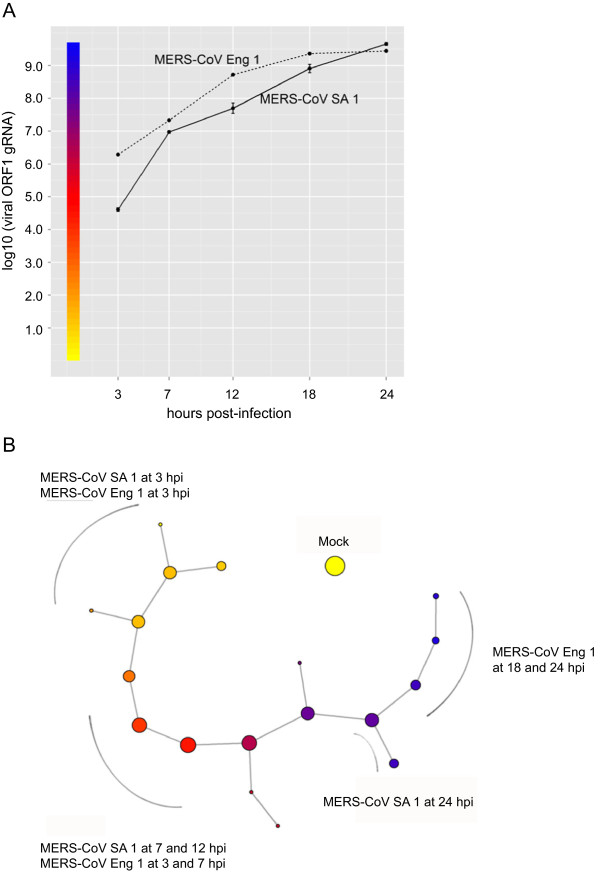
**Human airway epithelial cell culture model of MERS infection and the kinetics of the host response. A**. Calu-3 2B4 cells were infected with either MERS-CoV SA 1 or MERS-CoV Eng 1 (MOI 5). Cell lysates were harvested at 3, 7, 12, 18 and 24 hpi and viral genomic RNA measured by qRT-PCR using forward primer (KL200F) and reverse primer (KL201) spanning the CoV genome (Additional file 1: Table S5). The error bars represent the standard deviation among triplicate samples. **B**. Clustering graph for samples according to their Euclidean distances in gene expression obtained by an integration of viral genomic RNA levels. Samples with similar viral genomic RNA levels were grouped together. Using statistical criteria on the single linkage heights between all samples, we extracted the highly interconnected part (with smaller linkage heights). These samples were compared to highly interconnected samples of a second group of samples with overlapping viral genomic RNA levels of the first group and so forth. Whenever two highly interconnected parts had at least one sample in common we defined the two groups as adjacent. In the clustering graph, adjacent sample groups are linked by an edge, the node color represents the average viral genomic RNA levels of each sample group. Edge length or distance between nodes in the graph does not recapitulate spatial closeness of samples.

To further assess the magnitude impact of MERS-CoV infection in Calu-3 2B4 cells, we integrated viral genomic RNA levels into a topological assessment of host response differences. MERS-CoV genomic RNA was measured by qRT-PCR using primer (KL200F) located in the leader region (nt 9-28) and primer (KL201) located in the ORF1 region of the CoV genome (Figure 1A and Additional file 1: Table S5). Additionally, we measured viral titers by plaque assay and found MERS-CoV SA 1 had more robust infectious viral particle production than MERS-CoV Eng 1, with significantly different viral titers at 18 and 24 hpi, despite similar viral genomic RNA levels at these late time points (Additional file 2: Table S4). In measuring viral genomic RNA and infectious viral particle production, we assessed two different stages in the viral life cycle and the data support differences in the kinetics of replication for the two MERS-CoV strains. Since our analysis focuses on the host response to infection and its origin in pathogen recognition and activation of innate immune responses, we computationally integrated viral genomic RNA with host mRNA to better assess the differences in host response kinetics. Towards this end, we used a filtered clustering method [17] that reduced the space of 93 samples to a clustering graph of 18 nodes. First, the viral genomic RNA levels were used as a filter to bin the samples into overlapping subsets with similar viral load (i.e., viral gRNA). Second, within each subset, samples that showed a high degree of interconnection were retained. Thus, there was high similarity in gene expression levels within each subset. For a graphical representation, each set of retained samples formed a node and nodes were considered as adjacent whenever they had at least one sample in common (Methods and Figure 1B). Mock-infected samples were all in one isolated cluster. With increasing viral load, the temporal delay between MERS-CoV Eng 1 and MERS-CoV SA 1 started as early as 3 hours post infection (hpi), with 3 and 7 hpi MERS-CoV Eng 1 samples clustering with 7 and 12 hpi MERS-CoV SA 1 samples. Genomic RNA levels at 18 and 24 hpi between the two viruses were not statistically different as determined by the non-parametric Mann-Whitney test. Despite similar viral gRNA at 18 and 24 hpi (Figure 1A), the filtered clustering approach demonstrated MERS-CoV Eng 1 and MERS-CoV SA 1 were distinct at the late time points, with 18 and 24 hpi MERS-CoV Eng 1 samples separated from 24 hpi MERS-CoV SA 1 samples (Figure 1B).

Taken together, these whole transcriptome topological analyses showed robust differences in both kinetics and magnitude of the host response between MERS-CoV SA 1 and MERS-CoV Eng 1, despite similar viral replication. Another example where viral replication may not be the best readout of host response differences is icSARS-CoV. The deletion of the entire ORF6 gene of icSARS-CoV (icSARS-CoV ΔORF6) does not impact viral replication compared to wild-type icSARS-CoV, yet substantial differences in the host response between the two viruses are detected in Calu-3 2B4 cells [18].

### MERS-CoV SA 1 and MERS-CoV Eng 1 elicit striking differences in epithelial adherens junction signaling and pro-inflammatory cytokine signaling late in infection

**Figure 2:**
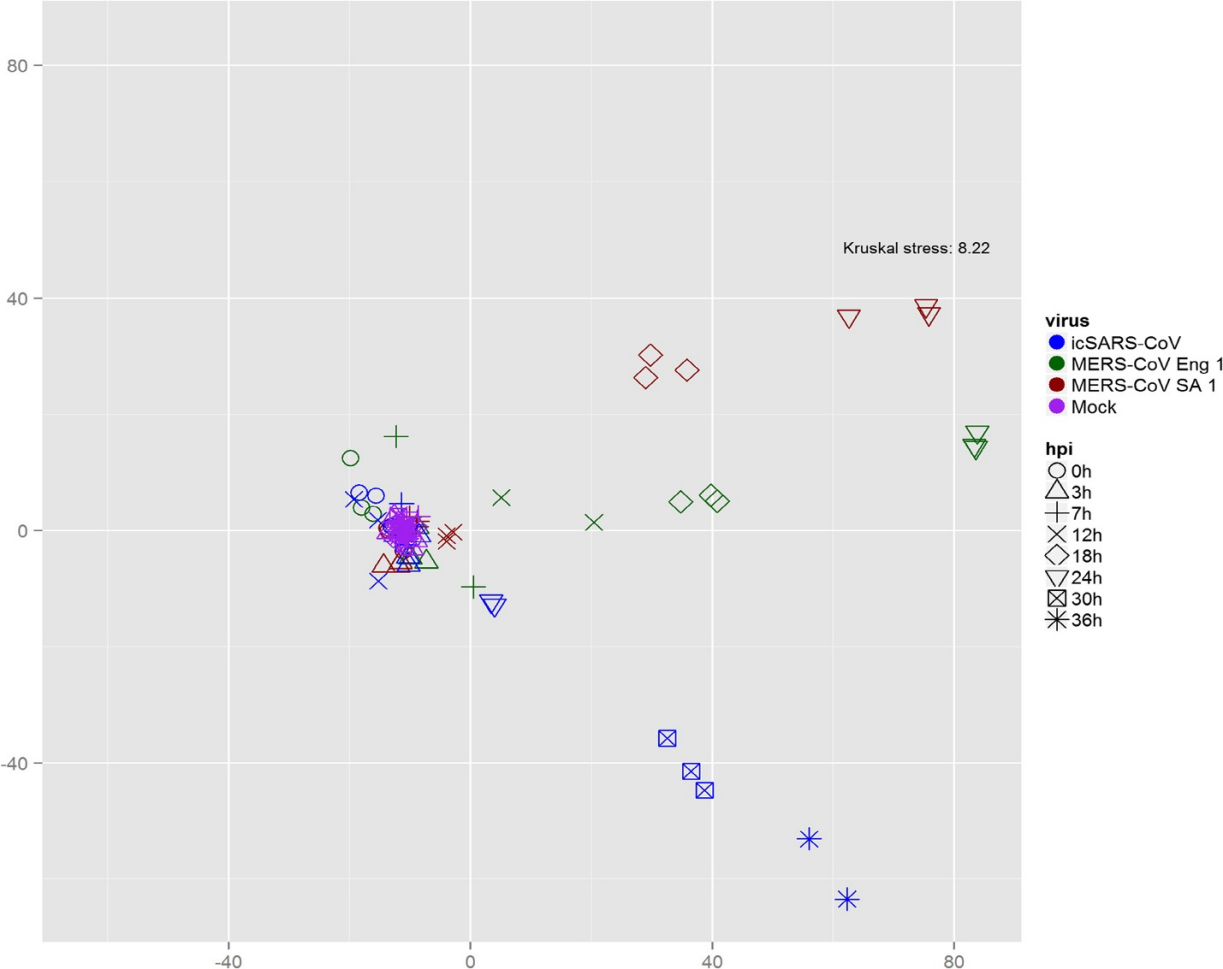
**Multidimensional scaling representation for Calu**-**3 MERS**-**CoV and SARS**-**CoV infections.** Calu-3 2B4 cells were infected with either MERS-CoV SA 1 or MERS-CoV Eng 1 (MOI 5) and cell lysates were harvested at 0, 3, 7, 12, 18 and 24 hpi. Calu-3 2B4 cells were infected with icSARS-CoV (MOI 5) and cell lysates were harvested at 0, 3, 7, 12, 24, 30 and 36 hpi. Samples are clustered based on 6432 DE genes changing in at least one virus condition and at one time-point The quality of the representation is provided by the Kruskal Stress criteria, with the relatively low percentage of Kruskal stress (8.22%) suggesting a faithful 2D representation of the statistically observed transcriptional differences between MERS-CoV SA 1, MERS-CoV Eng 1 and icSARS-CoV.

Differential gene expression between MERS-CoV Eng 1 and MERS-CoV SA 1 was directly examined by comparing fold changes with respect to time- and experiment-matched mocks (absolute log2 FC > 1, FDR-corrected *p*-value < 0.05). We found the most robust transcriptional changes to MERS-CoV SA 1 and MERS-CoV Eng 1 infections occurred at the late times points (18 and 24 hpi). Compared to MERS-CoV SA 1 there was an accelerated host response to MERS-CoV Eng 1, with differential gene expression observed as early as 7 hpi. As shown in Figure 2, MDS representation of MERS-CoV SA 1, MERS-CoV Eng 1 and icSARS-CoV samples for 6432 DE genes changing in at least one virus condition and at one time-point confirmed the early onset of host responses to MERS-CoV Eng 1 infection and the sustained separation between MERS-CoV Eng 1 and MERS-CoV SA 1 beginning at 18 hpi. The MDS representation also showed MERS-CoV Eng 1 samples in an intermediate position between MERS-CoV SA 1 and icSARS-CoV, with the host response to icSARS-CoV being largely delayed until 30 and 36 hpi. There is increasing evidence that interferon (IFN) antagonism and avoidance of interferon-stimulated gene (ISG) effector functions are major contributors to the delayed host response in icSARS-CoV infection [18, 19]. In this context, the intermediate positioning of MERS-CoV Eng 1 between MERS-CoV SA 1 and icSARS-CoV led us to investigate MERS-CoV strain-specific differences in innate immune signaling early in infection and the subsequent impact on the later host response.

**Figure 3:**
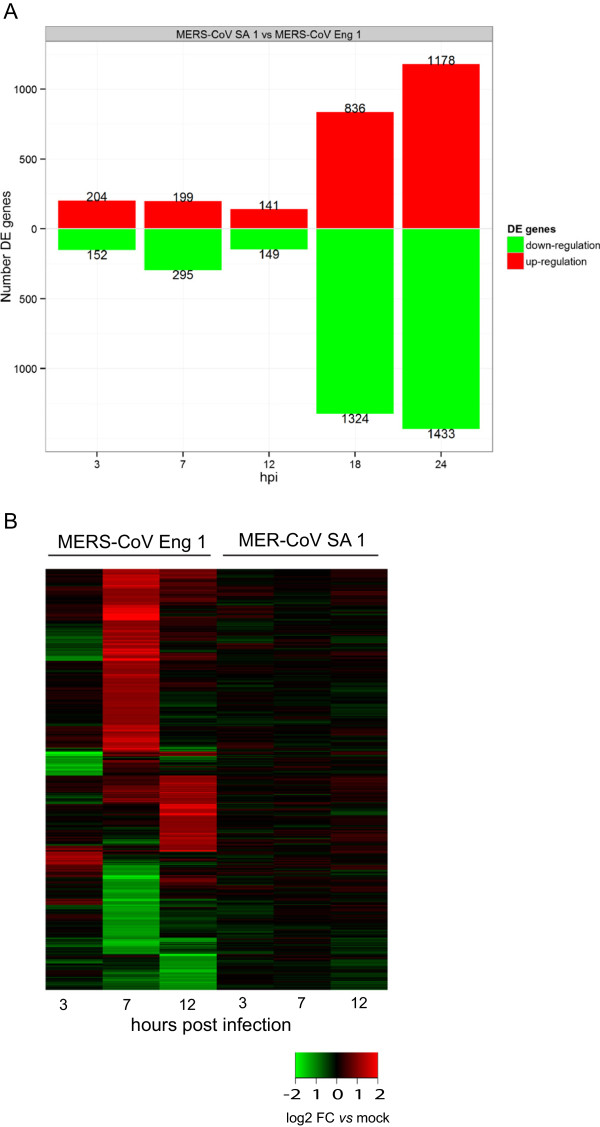
**Differentially expressed genes following MERS**-**CoV SA 1 and MERS**-**CoV Eng 1 infections. A**. Statistically significant DE genes between MERS-CoV SA 1 and MERS-CoV Eng 1 were identified at 3, 7, 12, 18 and 24 hpi (absolute log2 FC > 1, FDR-corrected *p*-value < 0.05). The bargraph shows the number of up-regulated and down-regulated DE genes between MERS-CoV SA 1 and MERS-CoV Eng 1 at each time point. **B**. Heatmap of differentially expressed genes following MERS-CoV SA 1 and MERS-CoV Eng 1 infection shows more than four hundred genes uniquely expressed in MERS-CoV Eng 1 infected cells at early time points (between 3 and 12 hpi), with differential expression criteria of absolute log2 FC > 1 against time- and dataset-matched mocks, FDR-corrected *p*-value < 0.05).

The greatest number of gene expression differences between MERS-CoV SA 1 and MERS-CoV Eng 1 were induced at the late time points, with 2160 genes and 2611 genes differentially expressed (DE) at 18 and 24 hpi, respectively (Figure 3A). There was a total of 4861 DE genes between MERS-CoV SA 1 and MERS-CoV Eng 1 in at least one time point (herein known as contrasting genes). In comparison to MERS-CoV SA 1, there was an earlier host response to MERS-CoV Eng 1 at 7 and 12 hpi. The accelerated Calu-3 response to MERS-CoV Eng 1 may be the result of the difference in kinetics of viral gRNA replication, with MERS-CoV Eng 1 more efficiently replicating at 3 and 7 hpi compared to MERS-CoV SA 1 (Figure 1A). Alternatively, the delay in the host response to MERS-CoV SA 1 may be due to the virus more efficiently evading innate immune responses that leads to enhanced viral replication compared to MERS-CoV Eng 1 (Additional file 2: Table S4). Between the two viruses there are amino acid differences in ORF4a and PLpro, viral proteins known to modulate the innate immune response during MERS infection [5–7]. We further examined host gene expression at the early time-points (3, 7 and 12 hpi) and found significant enrichment of genes associated with the STAT3 pathway. STAT3 pathway genes *CDC25A*, *MYC*, *SOCS3* and *SOCS4* were more strongly induced by MERS-CoV Eng 1 compared to MERS-CoV SA 1, particularly at 7 hpi (Figure 3B). Increased SOCS gene expression and decreased expression of *PIM1* gene in response to MERS-CoV Eng1 indicated decreased STAT3 activity and possibly differential induction of apoptosis-related pathways. In a direct virus comparison, differential expression of pro-apoptotic *BID*, *BAX*, and *BIM* genes was observed at the early time-points and by 24 hpi there was extensive cytopathic effects caused by both viral infections that was likely the result of caspase-dependent apoptosis, as previously shown for MERS infection [20]. Of the 4861 contrasting genes, 2653 genes were also DE against time- and data set-matched mocks. Hierarchical clustering of the 4861 DE genes resulted in distinct gene clusters, with striking expression pattern contrasts between MERS-CoV SA 1 and MERS-CoV Eng 1 at 18 and 24 hpi. As shown in Table 2, functional analysis of the five most prominent clusters with contrasting gene expression revealed enrichment of integrin linked kinase (ILK) signaling and epithelial adherens junction signaling pathways, glutathione metabolism, and interferon and pro-inflammatory signaling pathways. Genes related to glutathione metabolism included *GSTM1* and *GSTM3*, which were strongly downregulated in response to both viruses. ISGs, *IFIT1* and *IFIT3*, were highly induced in response to both MERS-CoV Eng 1 and MERS-CoV SA 1, with pronounced early up-regulation specifically in response to MERS-CoV Eng 1. Genes associated with ILK signaling and epithelial adherens junction signaling pathways were strongly downregulated in response to MERS-CoV SA 1, whereas MERS-CoV Eng 1 predominantly up-regulated expression of these genes at the late time points (Additional file 3: Figure S1). Within the highly enriched pathways, cellular genes including *PVRL1*, *RHOF* and *CREBBP* with highest expression contrasts between the two infections were chosen for confirmation by qRT-PCR (Additional file 4: Figure S2). Pro-inflammatory *CSF3* gene was found more highly induced by MERS-CoV SA 1 (log ratios in MERS-CoV SA1 and MERS-CoV Eng1 respectively: 2.2 and 0.5), whereas *CCL5* gene was more highly induced by MERS-CoV Eng 1 (log ratios in MERS-CoV SA1 and MERS-CoV Eng1 respectively: 0.71 and 2.2). Examination of the aforementioned contrasting genes showed that only a small number of those were already differentially expressed at the early time-points. For example, expression of *RHOF* gene in response to MERS-CoV Eng 1 was increased 4-fold relative to mock at 12 hpi, whereas MERS-CoV SA 1 did not induce *RHOF* gene expression at this time-point. We therefore focused on differences in the host response mainly at the later time-points that had the highest number of contrasting genes.

**Table 2:** **Canonical Pathways enriched in human airway cells infected with MERS**-**CoV**

MERS virus	Canonical pathway ^a^	-log ***p***-val	mol ^b^ mol ^c^
***MERS***-***CoV Eng 1 vs MERS***-***CoV SA 1***	Role of Macrophages, Fibroblasts and Endothelial Cells in RA	5.80	94/342
***(4861 DEG)***	Tumoricidal Function of Hepatic Natural Killer Cells	3.77	13/27
	VDR/RXR Activation	3.66	29/88
	Wnt/β-catenin Signaling	3.63	53/175
	G-Protein Coupled Receptor Signaling	3.60	74/276
***Cluster 2***	Wnt/β-catenin Signaling	5.40	23/175
***(1108 DEG)***	NGF Signaling	5.11	17/122
	Epithelial Adherens Junction Signaling	4.89	20/154
	PPARα/RXRα Activation	4.68	22/200
	ILK Signaling	4.27	22/205
***Cluster 4***	Glutathione-mediated Detoxification	5.51	5/45
***(194 DEG)***	Antigen Presentation Pathway	4.89	5/42
	Ascorbate Recycling (Cytosolic)	3.69	2/13
	Glutathione Redox Reactions I	3.46	2/24
	Aryl Hydrocarbon Receptor Signaling	2.95	6/171
***Cluster 10***	Notch Signaling	3.17	6/43
***(648 DEG)***	1D-myo-inositol Hexakisphosphate Biosynthesis II (Mammalian)	1.79	3/28
	D-myo-inositol (1,3,4)-trisphosphate Biosynthesis	1.79	3/25
	Ephrin B Signaling	1.71	6/82
	Tumoricidal Function of Hepatic Natural Killer Cells	1.52	3/27
***Cluster 11***	Interferon Signaling	2.40	2/36
***(61 DEG)***	Phenylethylamine Degradation I	1.96	1/11
	IL-17A Signaling in Airway Cells	1.87	2/76
	BMP signaling pathway	1.74	2/86
	TGF-β Signaling	1.66	2/94
***Cluster 12*** ***(576 DEG)***	Differential Regulation of Cytokine Production in Macrophages and T Helper Cells by IL-17A and IL-17 F	3.24	4/18
	Role of Cytokines in Mediating Communication between Immune Cells	2.87	6/54
	Differential Regulation of Cytokine Production in Intestinal Epithelial Cells by IL-17A and IL-17 F	2.82	4/23
	G-Protein Coupled Receptor Signaling	2.71	14/276
	Agranulocyte Adhesion and Diapedesis	2.55	11/192

Ingenuity Pathway Analysis was used to determine significant Canonical Pathways for the entire heatmap of contrasting genes (MERS-CoV Eng 1 *vs* MERS-CoV SA 1) and for individual Clusters 2, 4, 10, 11, and 12 that had the most contrasting patterns of gene expression at 18 and 24 hpi.

^a^RA: Rheumatoid Arthritis.

^b^Number of molecules differentially expressed in the canonical pathway.

^c^Total number of molecules in the annotated canonical pathway.

### MERS-CoV Eng 1 and MERS-CoV SA 1 elicit differences in the timing of cytokine-mediated innate immune and pro-inflammatory responses in Calu-3 cells

While there was no difference in IFN- β gene expression detected between the two MERS-CoV strains, there was differential IFN-α2 and IFN- γ gene expression. Specifically, expression of IFN-γ was downregulated early after infection in MERS-CoV Eng 1-infected Calu-3 cells and up-regulated in MERS-CoV SA 1-infected Calu-3 cells. To some extent, this was also observed for IFN-α2 gene expression, which showed higher up-regulation in response to MERS-CoV SA 1 at 24 hpi, as confirmed by qRT-PCR (Additional file 4: Figure S2). In addition, IFN-λ1 and IFN-λ2 were DE relative to mock with high up-regulation at 18 and 24 hpi for both viruses.

**Figure 4:**
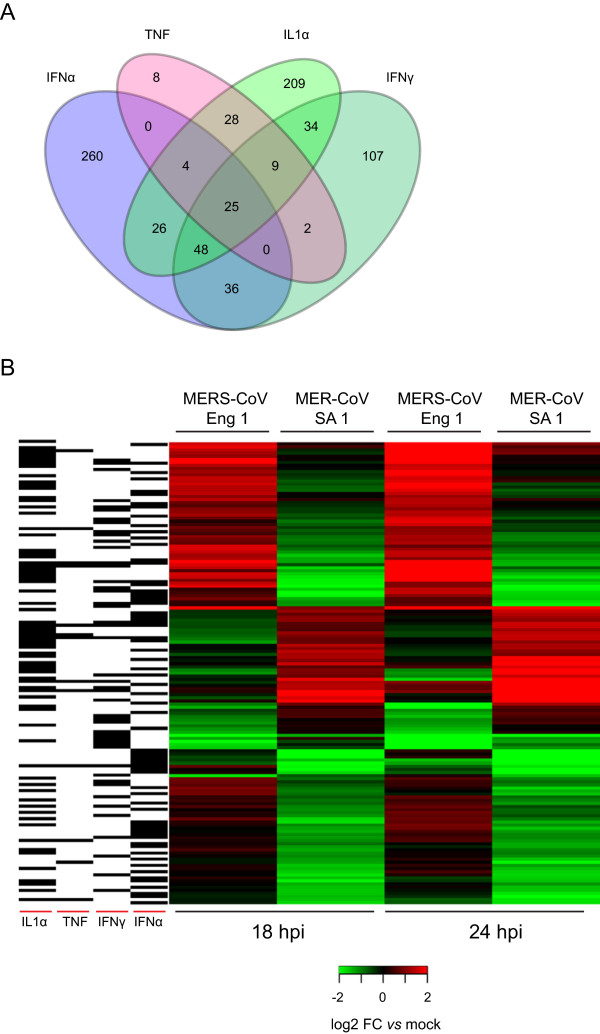
**Differential cytokine stimulated gene expression in human airway epithelial cells infected with distinct MERS**-**CoVs. A**. Venn diagram shows the number of genes which were DE after infection in any one virus or time point and their overlap within four sets of different cytokine stimulation. Whereas IFN-α, IFN-γ and IL-1α show a large number of specific genes, TNF stimulated DE genes share many genes with other cytokines **B**. Heatmap of 149 DE genes following either MERS-CoV SA 1 or MERS-CoV Eng 1 infection (MOI 5) that show strong contrasts at 18 and 24 hpi. The black bars on the left of the heatmap indicate whether genes were also DE after cytokine treatments (IFN-α, IFN-γ, IL-1α, TNF-α) in the same cell line system as the infection.

There were no virus-specific differences in expression of pro-inflammatory cytokines, IL-1α or TNFα-IP3, which were highly up-regulated in response to both MERS-CoV Eng 1 and MERS-CoV SA 1. The cell migration-promoting factor, TNFα-IP2 [21], was highly up-regulated for MERS-CoV Eng 1 alone (Additional file 4: Figure S2). Cytokines appear to be important for MERS infection. To explore potential mechanisms regulating cytokine activity in response to MERS-CoV, we designed a microarray experiment to analyze Calu-3 responses to various cytokine treatments and develop signatures that were then examined in the context of MERS-CoV-infected Calu-3 cells. Calu-3 cells were treated with either human recombinant interferon IFN-α, IFN-γ, TNF or IL-1α, and cell lysates collected at different time points post-treatment for microarray. We found 399 DE genes responsive to recombinant IFN-α and 261 DE genes responsive to recombinant IFN-γ, and as expected, the majority of these genes were up-regulated following stimulation [22]. In response to pro-inflammatory cytokine treatment, we found 76 DE genes responsive to recombinant TNF and 383 DE genes responsive to recombinant IL-1α, which were also differentially expressed in response to MERS-CoV SA 1 or MERS-CoV Eng 1. Many of these genes showed cytokine-specific expression for IFN-α (260 DE genes), IFN-γ (107 DE genes), and IL-1α (209 DE genes (Figure 4A). TNF induced the least number of DE genes compared to the other cytokines (8 DE genes). Within the cytokine-stimulated genes we identified a set of 149 genes showing strong contrasts at late time points (18 and 24 hpi) between MERS-CoV SA 1 and MERS-CoV Eng 1 (Figure 4B).

Topological analysis of gene expression restricted to IFN and pro-inflammatory cytokine genes sets revealed IFN induction (score: 0.74) as a major contributor to kinetic differences between MERS-CoV SA 1 and MERS-CoV Eng 1 infection (Table 1). The relative numbers of cytokine genes, which were also DE after MERS-CoV SA 1 or MERS-CoV Eng 1 infection (22% for IFN-α, 31% for IFN-γ, 31% for IL-1α and 45% for TNF), indicated the contribution of these specific gene sets to global gene expression changes in MERS infection. Functional analysis of the cytokine DE genes showed antigen presentation pathway was significantly enriched in MERS-CoV-induced IFN-γ responses, as well as enrichment of genes associated with adaptive immune responses such as OX40 signaling and cytotoxic T lymphocyte-mediated apoptosis pathways. IFN-α response genes were significantly enriched for cellular pathways involved in the antiviral response, such as interferon regulatory factor (IRF) activation by pathogen recognition receptors (PRRs). Pro-inflammatory mediators, IL-1α and TNF, which stimulate genes related to IL-17 signaling, were identified following MERS infection. For example, pro-inflammatory cytokine genes, *CXCL1*, *CCL2*, and *CCL20*, regulated by IL-17A [23] and play a prominent role in airway inflammation and disease, and NF-κB genes, *NFKBIA*, *NFKB2* and *NFKBIE*, were all found to be DE in response to TNF and MERS-CoV infection.

### Predicted role for STAT3 in mediating gene expression changes between MERS-CoV Eng 1 and MERS-CoV SA 1 in Calu-3 infected cells

To further investigate potential regulators mediating the gene expression contrasts between MERS-CoV Eng 1 and MERS-CoV SA 1 at the late time points, we performed an Upstream Regulator Analysis in Ingenuity Pathway Analysis (IPA) that identifies upstream transcriptional regulators based on the observed gene expression changes in the experimental data set and compiled knowledge of reported relationships between regulators and their known target genes within the Ingenuity Pathway Knowledge Base. The *NUPR1* gene encoding a transcription factor known as stress-activated nuclear protein 1 had a predicted inhibition (z-score of -2.373) for MERS-CoV SA 1 and predicted activation (z-score of 3.226) for MERS-CoV Eng 1 at 18 hpi (Table 3). This LPS-induced transcription factor is an important regulator of apoptosis, cell proliferation and autophagy [24–26]. Differentially expressed genes between MERS-CoV SA 1 and MERS-CoV Eng 1 that are binding partners and downstream targets, such as *EP300* and *TESK1*, shown in Additional file 3: Figure S1, were downregulated in MERS-CoV SA 1 virus-infected cells at 18 and 24 hpi and up-regulated in MERS-CoV Eng 1 virus-infected cells at these late time points, corroborating the predicted *NUPR1* regulator status in response to these two MERS-CoV strains.

**Table 3:** **Upstream regulators with predicted Activation and Inhibition states distinguishing MERS**-**CoV Eng 1 and MERS**-**CoV SA 1 infections**

Upstream regulator	p-value of overlap	MERS-CoV SA 1_18h	MERS-CoV Eng 1_18h	MERS-CoV SA 1_24h	MERS-CoV Eng 1_24h
**STAT3**	3.82E-14	-0.253	1.958	0.364	2.559
CTNNB1	1.20E-13	-1.187	1.465	-1.998	1.515
**ESR1**	1.82E-12	0.519	-0.728	-0.21	-1.083
NFKBIA	2.30E-10	0.604	1.108	0.635	1.477
NUPR1	2.40E-10	-2.373	3.226	-0.347	2.705
**SP1**	4.13E-10	-2.222	2.021	-1.62	0.831
NR3C1	5.66E-10	-2.65	-0.895	-1.582	-1.658
**CREB1**	1.86E-09	-2.138	2.52	-1.085	1.582
CEBPA	3.63E-09	0.39	-1.513	0.075	-1.162
**JUND**	3.71E-09	1.248	0.282	0.443	-0.201
HIF1A	1.32E-08	-0.442	0.622	-0.801	1.993
**TP53**	1.89E-08	-1.46	0.719	-1.608	1.228
**AHR**	2.57E-08	1.482	1.642	0.227	0.984
**FOS**	2.64E-08	0.188	0.188	0.188	1.076
FOXO3	2.76E-08	0.086	0.685	-0.198	-0.457
Histone h3	2.81E-08				
**CEBPB**	6.11E-08	0.795	-0.929	-0.242	0.845
TP63	6.62E-08	-0.084	1.834	-0.557	0.605
**NFKB1**	1.35E-07	-0.025	0.564	0.93	0.867
NFATC3	1.52E-07	-1.082	0.8	-0.687	-0.286
PDX1	1.95E-07	1.223	1.206	1.077	1.673
SMARCA4	2.10E-07	-1.283	0.435	-0.377	1.115
EGR1	2.16E-07	0.38	1.407	-0.626	0.464
Ap1	5.52E-07				
CREM	6.36E-07	-0.951	1.412	-0.254	2.437
FOXL2	6.57E-07	1.267	2.428	0.682	3.559

Ingenuity Pathway Analysis was used to determine the top 20 Upstream Regulators. z-scores for predicted upstream regulators (|z| >2) at each time point are shown. z >2 predicts activation of the upstream regulator. z < -2 predicts inhibition of the upstream regulator. The Activation z-score was used to rank the Upstream Regulator based on the “Inhibited” status (darker cells indicated more significant “Inhibited” status of Upstream Regulator).

Upstream Regulators in bold indicates enrichment of DNA-binding motifs (human) via TRANSFAC promoter analysis intersected DE genesets for MERS-CoV Eng 1 and MERS-CoV SA 1 at either 18 or 24 hpi (*q*-value of 10^-7^).

**Figure 5:**
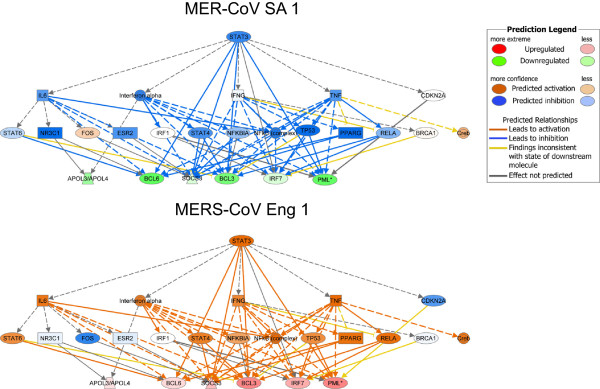
**STAT3 is a predicted regulator mediating the gene constrasts between MERS**-**CoV Eng 1 and MERS**-**CoV SA 1.** Upstream Regulator Analysis in IPA was used to predict regulators and infer their activation state based on the literature and gene expression of target genes in the data set. STAT3 is the master regulator of a small causal network that postulates differential STAT3 activity in MERS-Co-V SA 1 and MERS-CoV Eng 1 infections. STAT3 activity affects a number of other regulators that explains the downstream gene expression changes in the data set. The color of the lines (edges) signifies the expected direction of effect between two nodes. Blue represents predicted inhibition and orange represents predicted activation. Yellow signifies inconsistency between the gene expression in the data set and the annotated relationship. The color of the node signifies the z-score calculated from the data set. Blue: z-score < -2 and orange: z-score > 2. The downstream genes show gene expression in infected cells relative to mock-infected cells. Green: down-regulated expression and red: upregulated expression).

We also identified STAT3 as a predicted upstream regulator at 24 hpi in response to MERS-CoV Eng 1 (z-score = 2.559), with up-regulation of downstream target genes including *BCL6*, *SOCS3*, *BCL3*, *IRF7* and *PML* (Figure 5, Table 3). Through a separate binding motif prediction analysis using JASPAR and PSCAN, we confirmed the STAT3 prediction based on enrichment of STAT3 binding sequences in DE genes at the late time points (Table 3). Conversely, MERS-CoV SA 1 infection resulted in downregulation of these same target genes, suggesting a predicted inhibition or delay in STAT3 regulator activity, though STAT3 did not reach a statistically significant z-score (z-score = 0.364). Among the STAT3 target genes *BCL3* had the highest contrasting expression between the viruses. The *BCL3* gene was highly up-regulated in response to MERS-CoV Eng 1 (FC = 1.5) and downregulated in response to MERS-CoV SA 1 (FC = -1.66) at 18 hpi. qRT-PCR analysis of *BCL3* mRNA expression, and several additional STAT3 target genes, including *BCL6*, *PML*, and *IRF7*, were in agreement with the microarray findings (Additional file 4: Figure S2). Among these genes, IRF7 plays an important role in the cellular antiviral response and Faure and colleagues reported high levels of *IRF7* gene expression in BAL cells from a patient that recovered from MERS infection [12].

STAT3 acts as a critical regulator of cellular repair processes upon acute lung injury and *BCL3* has been shown to reduce lung inflammation in mice by regulating granulocytes [27]. In addition to *BCL3*, several other contrasting STAT3 target genes, including *BCL6*, *IL11*, and *PML*, have been reported to impact pro-inflammatory responses [28–30], epithelial integrity and the severity of lung injury after infection [27, 31]. We postulate the difference in pro-inflammatory cytokine gene expression may be the result of differential STAT3 activity. For example, SARS-CoV directly impacts STAT3 activity by dephosphorylating STAT3 at Tyr-705 after 18 hpi in infected Vero E6 cells [32]. Here, the MERS-CoV strain-specific differences may be a causative factor in leading to differential STAT3 activation and the down-stream effects of pro-inflammatory responses.

## Conclusion

MERS-CoV is a novel pathogenic coronavirus and the innate and inflammatory response during MERS infection is poorly understood. Using an established human airway culture model we found differences in host gene expression between MERS-CoV SA 1 and MERS-CoV Eng 1 that support the hypothesis of strain-specific differences related to functional differences in the sensitivity to innate immune responses [4]. Through our genomics-based approach, we found (i) topological differences in the kinetics and magnitude of the host response to MERS-CoV SA 1 and MERS-CoV Eng 1, with (i) a precursory host response in MERS-CoV Eng 1-infected cells, (ii) differential expression of innate immune and pro-inflammatory responsive genes between MERS-CoV Eng 1 and MERS-CoV SA 1 that may be associated with downstream effects of IFN, TNF and IL-1α signaling, and (iii) a predicted activation for STAT3 mediating gene expression relevant for epithelial cell remodeling in MERS-CoV Eng 1 infection.

At present, nonhuman primates serve as the best available model of MERS-CoV pathogenesis, with animals developing moderate clinical disease and signs of histopathologic changes in the lung [33, 34]. While there is no current small-animal model of MERS infection, immunocompromised mice transduced with an adenoviral vector expressing human DPP4 show increased susceptibility to MERS-CoV infection [35]. In a separate study, a variant of *Pipistrellus* bat coronavirus (BtCoV) strain HKU5 expressing the SARS-CoV spike (S) glycoprotein ectodomain (BtCoV HKU5-SE) resulted in enhanced morbidity and acute changes in lung histopathology in aged BALB/c mice following mouse adaptation [36]. The present cytokine systems approach provides valuable insight into differences of cellular antiviral responses to distinct MERS-CoV strains. In line with these observations, reversal of infection gene signatures that can attenuate viral replication or enhance innate immune responses to the most highly pathogenic MERS-CoV strain could be investigated in this model system. We are beginning to better understand that different MERS-CoV strains can result in variable host responses, as observed with the recent clinical study by Faure and colleagues [12]. The patients infected with MERS-CoV SA 1 and MERS-CoV Eng 1 had both a fatal outcome, with the MERS-CoV SA 1-infected patient succumbing to infection within 11 days after admission, 18 days to death following initial symptoms [1]. The MERS-CoV Eng 1 patient on the other hand had an initially transient illness and then rapidly declined to a severe acute respiratory distress syndrome remaining in this state for 9 months before death [2]. Having an efficient innate immune response likely dictates a patient’s disease progression and would thus be a primary goal for *in silico* drug predictions, which could then be tested *in vitro* with a particular focus on cytokine stimulated genes [37]. Toward this end, extending the collection of transcriptomic profiles to include more MERS-CoV strains will be important for a deeper view of the host response during MERS infection and toward a greater understanding of MERS-CoV pathogenicity.

## Methods

### Cells and viruses

Calu-3 2B4 cells were cultured in minimal essential media (MEM; Gibco) supplemented with 20% fetal bovine serum (HyClone) and 1% antibiotic antimycotic (Gibco). Human coronavirus MERS-CoV SA 1 (GenBank: JX869059.2) was received from Bart L. Haagmans (Erasmus Medical Center, Rotterdam, Netherlands). MERS-CoV SA 1 was isolated from the sputum of a 60-year-old man in Saudi Arabia who died after developing acute respiratory distress syndrome (ARDS) in June 2012 [1]. Human coronavirus MERS-CoV Eng 1 (GenBank: KC667074.1) was received from the United Kingdom Health Protection Agency (HPA) Imported Fever Service. MERS-CoV SA 1 and MERS-CoV Eng 1 were propagated in VeroE6 cells and supernatants clarified. For questions of written informed consent for participation in the study, we refer to [1] and [2], where isolation of viruses from clinical samples is described. Virus stocks were tittered on VeroE6 cells using standard methods, as previously described [14]. SARS-CoV experiments were derived from the infectious clone of SARS-CoV (icSARS-CoV) as described in [18, 38].

### Calu-3 2B4 infections

All work was performed in a biosafety level 3 (BSL3) facility at the University of North Carolina-Chapel Hill. Cells were washed with phosphate-buffered saline (PBS) and inoculated with virus at a multiplicity of infection (MOI) of 5 plaque-forming unit (PFU) per cell or mock diluted in PBS for 40 min at 37°C. Following inoculation, cells were washed 3 times, and fresh medium was added. Triplicate infected Calu-3 2B4 cultures and triplicate time-matched mock-infected controls were harvested at 0, 3, 7, 12, 18 and 24 hpi.

### Cytokine treatment of Calu-3 cells

Calu-3 cells were treated with either IFN-α (10 ng/ml) (Sigma I4276), IFN-γ (500 U/ml) (Sigma I32265), IL-1α (0.001 ng/ml) (Peprotech, #200-01A) or TNF (0.05 ng/ml) (Peprotech, #300-01A) in triplicate for each cytokine. Treated cell lysates (*n* = 3) and time-matched mock-treated cell lysates (*n* = 3) were harvested at 0, 3, 6, and 18 hr following IFN-α treatment, at 0, 3, 6, and 21 hr following IFN-γ treatment, at 3, 6, and 24 hr following IL-1α treatment, and at 6 hr following TNF treatment. Total RNA was extracted from each sample and gene expression analyzed by microarray. Probe labeling and microarray slide hybridization for pooled replicates for each treatment per time-point was performed using Human Genome CGH Microarray (G4447A; Agilent Technologies).

### RNA isolation and microarray processing

RNA isolation from Calu-3 2B4 cells infected with MERS-CoV SA 1 and subsequent hybridization to Agilent 4 × 44K human HG arrays was previously reported in [11]. RNA isolation from Calu-3 2B4 cells infected with MERS-CoV Eng 1 was performed following the same protocol used for MERS-CoV SA 1-infected Calu-3 samples and used for subsequent hybridization to Agilent 8 × 60K human arrays. Calu-3 2B4 microarray experiments with an infectious clone recombinant SARS-CoV (icSARS-CoV) (AY278741) was reported in [18]. Using Agilent QC criteria we removed one 12 hpi replicate infected with MERS-CoV SA 1 and one 7 hpi replicate infected with MERS-CoV Eng 1. Due to the absence of mock samples at 24 hpi in the MERS-CoV Eng 1, we used the mock samples harvested at 18 hpi for the comparison at 24 hpi. In order to merge MERS-CoV Eng 1 and MERS-CoV SA1 data sets, we considered only probes that were mapped to gene names (Refseq IDs) in both arrays. To remove batch effects from the merged data sets we applied the R function ComBat [39]. The resulting data set was then quantile normalized. For each sample, a log2 fold change value was calculated as a difference between log2 normalized data for each sample and the average of log2 normalized data for time- and data set-matched mock-infected samples. The same procedure, but handled as separate data sets, was applied to the cytokine treatment microarray data sets.

### Statistical analysis

Statistical analyses for viral genomic RNA and viral titer changes in Calu-3 2B4 cells infected with either MERS-CoV SA1 or MERS-CoV were performed by using two-sided non-parametric Mann-Whitney U test (Additional file 2: Table S4).

### Topology analysis

For normalized intensity data, the Euclidean distance matrix between samples was clustered with respect to sample-matched viral genomic RNA measurements using an algorithm described in [17]. We used a freely available MATLAB implementation of this algorithm (http://appliedtopology.org/) with the parameter setting filterSamples = 5, pverlapPct = 65, mFudge = 5. The topology induced by a distance matrix can be described by a filter function, which assigns to every sample a value (phenotypic outcome, viral load, etc.). First, the topological space is decomposed into overlapping subsets using level sets of the filter function. The set of samples falling into a particular subset are clustered, and the cluster tree is partitioned into two parts using statistical criteria of the single linkage length. Only the part of the cluster tree with small linkage lengths (dense part) is retained. If resulting filtered clusters of different subsets have at least one sample in common, then they are defined as adjacent in a global discrete cluster graph. Topological differences between MERS-CoV SA 1 and MERS-CoV Eng 1 gene expression during the course of infection was furthermore assessed using homology persistence barcodes (only 0- and 1-homology were taken into account) as described in [15]. For calculations, we used the freely available R package phom [40] and their maximum weighted bipartite graph matching [41] as a measure of difference (see R script implementation in Additional file 5). Additionally, multi-dimensional scaling (MDS) was performed on the Euclidean distance matrix between samples after differential gene expression statistical analysis [42].

### Differential gene expression analysis

Differential expression was determined by comparing MERS-CoV SA 1- and MERS-CoV Eng 1-infected replicates to time- and data set-matched mock-infected controls, based on a linear model fit for each probe using the R package Limma [43]. The same method was applied to determine differential expression between strains using time-matched MERS-CoV SA 1- and MERS-CoV Eng 1-infected samples. Criteria for differential expression were an absolute log2 fold change of 1 and a *q* value of <0.05 calculated using a moderated t test with Benjamini-Hochberg correction. The cytokine Calu-3 treatment data sets were analyzed separately using the same methods and cut-offs as the virus-infected Calu-3 cells. Differentially expressed genes were determined to be statistically significant in at least on time point and for at least one treatment.

### Quantitative reverse transcription PCR (qRT-PCR)

RNAs were reverse transcribed using the QuantiTect Reverse Transcription Kit (Qiagen). The resulting cDNA samples were diluted 50X and run on the 7900HT Fast Real-Time PCR System (Applied Biosystems) using Power SYBR Green PCR Master mix (Life Technologies) and 200 nM primers. Primer sets were designed using Primer3 [44]. The primer sequences for cellular gene targets and the strand-specific primers for quantifying viral genomic RNA are listed in Additional file 1: Table S5. For cellular gene mRNA quantification, relative gene expression in infected samples compared to that in mock-infected samples was calculated using the 2^-ΔΔ*CT*^ method [45]. The *RPL14* gene was selected as an internal control (calibrator) due to non-significant changes in *RPL14* gene expression in MERS-CoV SA 1- and MERS-CoV Eng 1-infected Calu-3 2B4 cells, as determined by microarray analysis.

### Functional enrichment analysis

Functional analysis of statistically significant gene expression changes was performed using Ingenuity Pathways Knowledge Base (IPA; Ingenuity Systems). For all gene set enrichment analyses, a right-tailed Fisher’s exact test was used to calculate *P*-values associated with each biological function and canonical pathway. Upstream Regulator Analysis in IPA was used to predict regulators and infer their activation state based on prior knowledge of expected effects between regulators and their known target genes according to the Ingenuity Knowledge Base (IKB). The calculated *z*-score signifies whether gene expression changes for known targets of each regulator are consistent with what is expected from the literature (*z* > 2, regulator predicted to be activated, *z* < -2, regulator predicted to inhibited). In addition to IPA Upstream Regulator Analysis, we also used EnrichR [46], a collaborative gene list enrichment tool, to determine possible upstream regulators via overrepresented transcription factor binding site motifs [47].

Causal Network Analysis in IPA was used to understand gene expression changes and causal relationships between genes and networks of upstream regulators in the experimental dataset. The genes within the causal network represent nodes and the edge that defines the biological relationship between two nodes is represented as an arrow signifying regulation. Dashed arrows represent indirect relationships and solid arrows represent direct relationships. All edges are supported by at least one published reference or from canonical information stored in IKB.

### Availability of supporting data

Raw microarray data have been deposited in NCBI’s Gene Expression Omnibus and are accessible through GEO accession GSE33267 (icSARS-CoV), GSE45042 (MERS-CoV SA 1), GSE56677 (MERS-CoV Eng 1), GSE33264 (IFN-α, and IFN-γ, Calu-3 treatments), and GSE56678 (IL-1α, and TNF Calu-3 treatments). All data sets and associated metadata have been submitted to Virus Pathogen Resource (ViPR, http://www.viprbrc.org). Study details can be accessed through the Systems Virology website (http://www.systemsvirology.org).

## Electronic supplementary material

Additional file 1: Table S5: Primer sequences used for qRT-PCR analysis of viral genomic RNA expression of MERS-CoV SA1 or MERS-CoV Eng1 and of gene expression changes in Calu-3 2B4 cells infected with either MERS-CoV SA1 or MERS-CoV Eng1. (DOC 36 KB)

Additional file 2: Table S4: MERS-CoV replication in human airway Calu-3 2B4 cells. (DOCX 15 KB)

Additional file 3: Figure S1: MERS-CoV SA 1 and MERS-CoV Eng 1 differentially regulate adherens junction genes facilitating cell-cell adhesion. Average log2 fold-change expression of 22 DE genes associated with ILK signaling and 20 DE genes associated with epithelial adherens junction signaling. Calu-3 cells were infected with MERS-CoV Eng 1 or MERS-CoV SA 1 (MOI = 5) and total cellular RNA was isolated at 18 and 24 hpi. Red indicates gene expression was increased relative to the time-matched mock-infected reference and green indicates gene expression was decreased relative to the time-matched mock-infected reference. Enrichment analysis of MERS-CoV contrasting genes from cluster 2 was performed using IPA. (PDF 466 KB)

Additional fie 4: Figures S2: Contrasting gene expression profiles of MERS-CoV-infected Calu-3 cells. Calu-3 cells were infected with MERS-CoV Eng 1 or MERS-CoV SA 1 (MOI = 5) and total cellular RNA was isolated at 18 and 24 hpi. Relative gene expression was calculated using the 2^-ΔΔ*Ct*^ method [45] and is shown as log2 fold-change of MERS-CoV-infected samples relative to *RPL14* endogenous control. Microarray gene expression for each cellular target is shown as log2 fold-change of MERS-CoV-infected samples relative to pooled mock-infected samples for a comparison with the mRNA expression profiles determined by qRT-PCR. (PDF 944 KB)

Additional file 5: **R implementation of maximal bipartite graph matching algorithm to calculate differences in persistence homology barcodes.** (TXT 2 KB)

## Abbreviations

MERS: Middle East respiratory syndrome
CoV: Coronavirus
ISG: Interferon stimulated gene
IFN: Interferon
RNA: Ribonucleic acid
gRNA: Genomic RNA
DE: Differentially expressed
HPI: Hours post-infection
MDS: Multidimensional scaling
IPA: Ingenuity Pathway Analysis
PRR: Pathogen recognition receptor
ARDS: Acute respiratory distress syndrome
PBS: Phosphate buffered saline
ORF: Open reading frame
MDA5: Melanoma Differentiation-Associated protein 5
RIG-I: Retinoic acid-inducible gene 1
NSP: Nonstructural protein
PLpro: Papain-like protease
PCR: Polymerase chain reaction
MOI: Multiplicity of infection
PFU: Plaque-forming unit
ILK: Integrin linked kinase.
