# Supplementary material for: Cytokine systems approach demonstrates differences in innate and pro-inflammatory host responses between genetically distinct MERS-CoV isolates
Source: BMC Genomics. 2014 Dec 22;15(1):1161. doi: 10.1186/1471-2164-15-1161 (PMC4522970; doi:10.1186/1471-2164-15-1161)
Supplement: Supplementary file 1 — Additional file 1: Table S5: Primer sequences used for qRT-PCR analysis of viral genomic RNA expression of MERS-CoV SA1 or MERS-CoV Eng1 and of gene expression changes in Calu-3 2B4 cells infected with either MERS-CoV SA1 or MERS-CoV Eng1. (DOC 36 KB) [file 12864_2014_7078_MOESM1_ESM.doc]

**Supplementary Table 5:** Primer sequences used for qRT-PCR analysis of viral genomic RNA expression of MERS-CoV SA1 or MERS-CoV Eng1 and of gene expression changes in Calu-3 2B4 cells infected with either MERS-CoV SA1 or MERS-CoV Eng1.

| **Target** | **Sense primer sequence (5’ to 3’)** | **Antisense primer sequence (5’ to 3’)** |
| --- | --- | --- |
| *CoV genome* | GAATAGCTTGGCTATCTCAC | CACAATCCCACCAGACAA |
| RPL14 | TTTCATCCTCAAGTTTCCGC | TTCAATCTTCTTGGCCCATC |
| BCL3 | CCGGAGGCGCTTTACTACC | TAGGGGTGTAGGCAGGTTCAC |
| BCL6 | AGCCACAAGACCGTCCATAC | CGAGTGTGGGTTTTCAGGTT |
| CREBBP | GTCGTGTGCAGTTCTTCCAA | TCAGAGACGAGAGCAAGCAA |
| EGR1 | ACCCCTCTGTCTACTATTAAGGC | TGGGACTGGTAGCTGGTATTG |
| IFNA2 | GCTTGGGATGAGACCCTCCTA | CCCACCCCCTGTATCACAC |
| PML | GGATGAAGTGCTACGCCTCG | TCCCCTGGGTGATGCAAGA |
| PVRL1 | CACCTGCAAAGCTGATGCTA | CGTCCCAGGTGAAGTCTCTC |
| RHOF | GGAAGGAGCTGAAGATCGTG | GGCCGTGTACTTCTCGAACA |
| TNFAIP2 | ACACCTACATGCTGCTGCTCT | ATACCCTGCAGCTCACCCA |
| IRF7 | GGGTGTGTCTTCCCTGGATA | GCTCCATAAGGAAGCACTCG |
