# Supplementary material for: Cytokine systems approach demonstrates differences in innate and pro-inflammatory host responses between genetically distinct MERS-CoV isolates
Source: BMC Genomics. 2014 Dec 22;15(1):1161. doi: 10.1186/1471-2164-15-1161 (PMC4522970; doi:10.1186/1471-2164-15-1161)
Supplement: Supplementary file 2 — Additional file 2: Table S4: MERS-CoV replication in human airway Calu-3 2B4 cells. (DOCX 15 KB) [file 12864_2014_7078_MOESM2_ESM.docx]

Supplementary Table S4: MERS-CoV replication in human airway Calu-3 2B4 cells

|  | **viral titer in Calu-3 2B4 cells (mean log_10_ PFU)** | | | | | | | **Viral genomic RNA** |
| --- | --- | --- | --- | --- | --- | --- | --- | --- |
| **hours post infection** | **MERS-CoV SA 1** | | | **MERS-CoV Eng 1** | | | **Mann-Whitney U test P value** | **Mann-Whitney U test P value** |
| 3 | 3.47 | 3.56 | 3.27 | 3.65 | 3.67 | 3.64 | 0.69 | 0.35 |
| 7 | 4.53 | 5.23 | 4.77 | 3.92 | 4.30 | 3.94 |  |  |
| 12 | 6.66 | 6.48 | 6.50 | 5.96 | 5.87 | 5.79 |  |  |
| 18 | 6.96 | 7.19 | 7.46 | 6.29 | 6.74 | 6.63 | 0.0043 | 1 |
| 24 | 7.59 | 7.67 | 7.52 | 6.79 | 7.00 | 6.96 |  |  |

Calu-3 2B4 cells were infected at an MOI of 5 with either MERS-CoV SA 1 or MERS-CoV Eng 1. Three replicate wells from each group were used to determine virus titers and viral genomic RNA levels. We applied the two-sided Mann-Withney U test on samples pooled for early time points (3,7, and 12 hpi) and late time points (18, 24 hpi). Titer data showed a significant location shift for late time points between MERS-CoV SA 1 and MERS-CoV Eng 1. For viral genomic RNA data there is no statistically significant difference at late time points.
