# Supplementary figures and images for: Cytokine systems approach demonstrates differences in innate and pro-inflammatory host responses between genetically distinct MERS-CoV isolates
Source: BMC Genomics. 2014 Dec 22;15(1):1161. doi: 10.1186/1471-2164-15-1161 (PMC4522970; doi:10.1186/1471-2164-15-1161)

Supplementary Figure 1

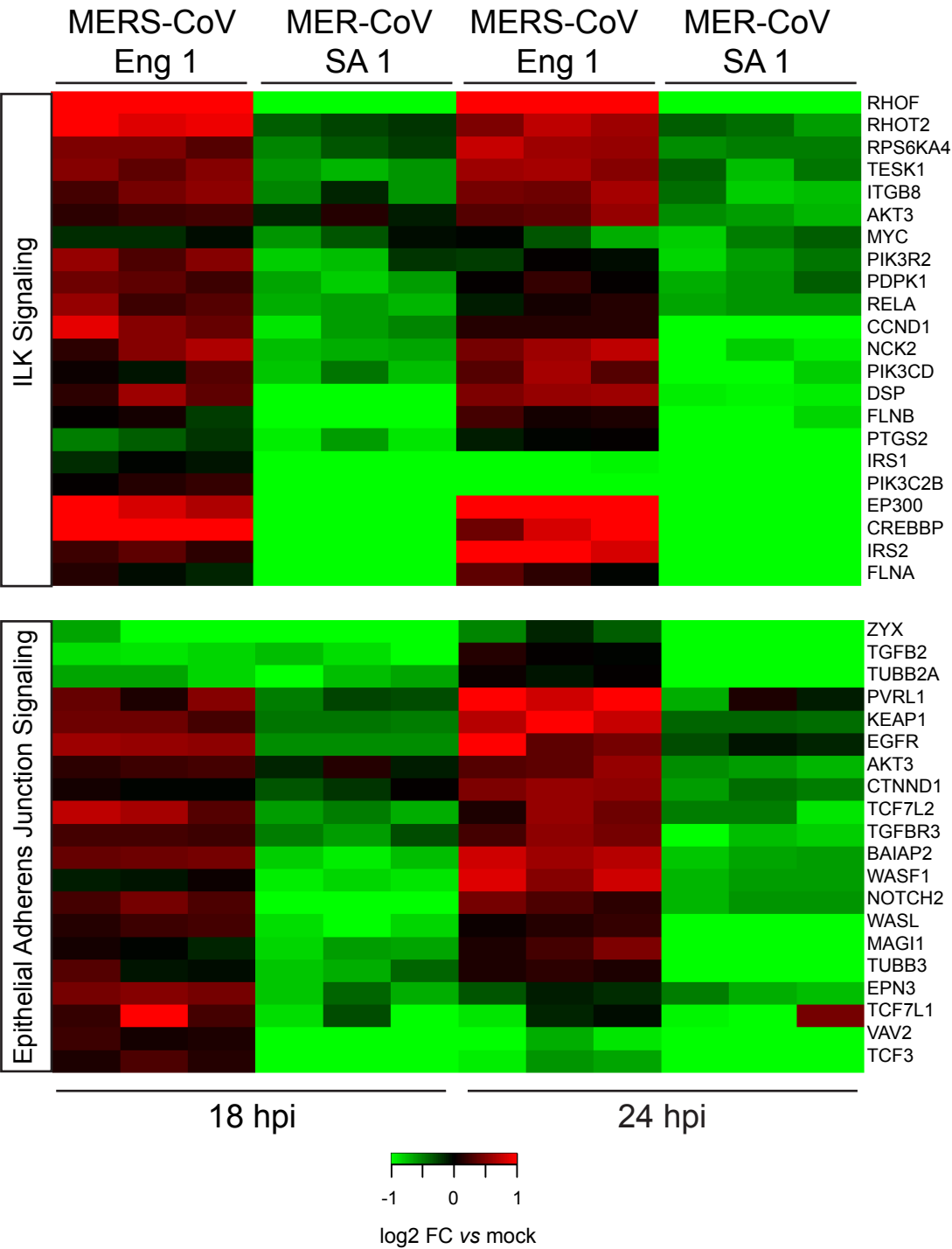

Supplement: Supplementary file 3 — Additional file 3: Figure S1: MERS-CoV SA 1 and MERS-CoV Eng 1 differentially regulate adherens junction genes facilitating cell-cell adhesion. Average log2 fold-change expression of 22 DE genes associated with ILK signaling and 20 DE genes associated with epithelial adherens junction signaling. Calu-3 cells were infected with MERS-CoV Eng 1 or MERS-CoV SA 1 (MOI = 5) and total cellular RNA was isolated at 18 and 24 hpi. Red indicates gene expression was increased relative to the time-matched mock-infected reference and green indicates gene expression was decreased relative to the time-matched mock-infected reference. Enrichment analysis of MERS-CoV contrasting genes from cluster 2 was performed using IPA. (PDF 466 KB) [file 12864_2014_7078_MOESM3_ESM.pdf]

# Supplementary Figure 2

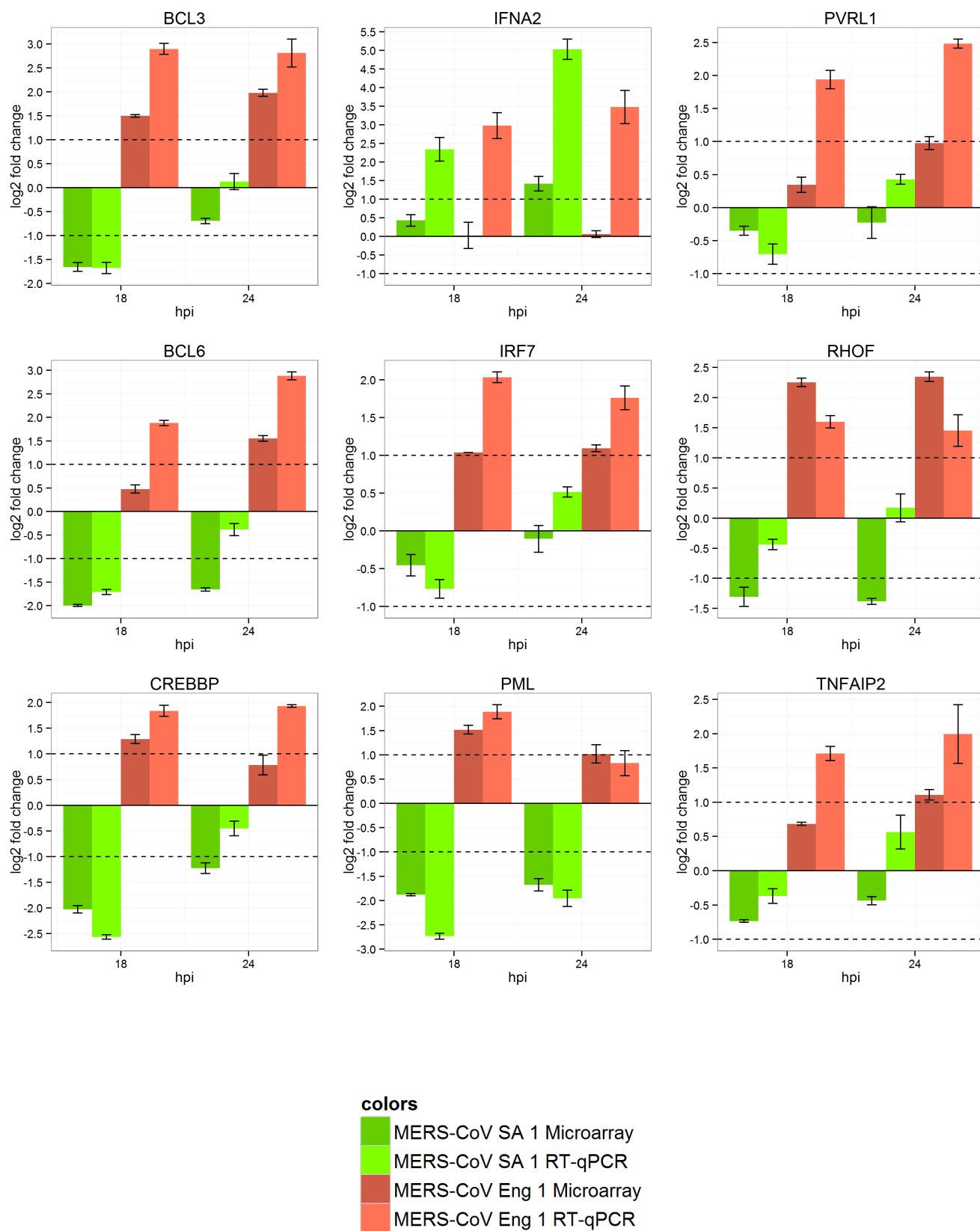

Supplement: Supplementary file 4 — Additional fie 4: Figures S2: Contrasting gene expression profiles of MERS-CoV-infected Calu-3 cells. Calu-3 cells were infected with MERS-CoV Eng 1 or MERS-CoV SA 1 (MOI = 5) and total cellular RNA was isolated at 18 and 24 hpi. Relative gene expression was calculated using the 2-ΔΔCt method [45] and is shown as log2 fold-change of MERS-CoV-infected samples relative to RPL14 endogenous control. Microarray gene expression for each cellular target is shown as log2 fold-change of MERS-CoV-infected samples relative to pooled mock-infected samples for a comparison with the mRNA expression profiles determined by qRT-PCR. (PDF 944 KB) [file 12864_2014_7078_MOESM4_ESM.pdf]
